# The Case for Regionalization: Standardizing the Hub-and-Spoke Model in Shock Care

**DOI:** 10.1016/j.jacadv.2026.102825

**Published:** 2026-05-18

**Authors:** Mukunthan Murthi, Tareq Alyousef, Aviral Vij

**Affiliations:** a Division of Cardiology, John H Stroger Hospital of Cook County, Chicago, Illinois, USA; b Division of Cardiology, Rush University Medical Center, Chicago, Illinois, USA

**Keywords:** acute myocardial infarction, cardiogenic shock, mechanical circulatory support, transfer

**Figure:**
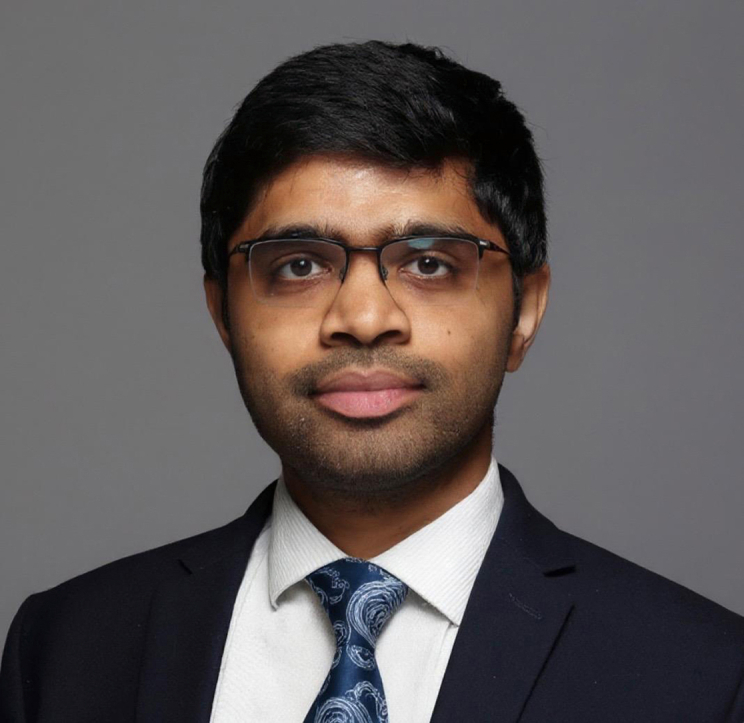

From the SHOCK trial to the DanGer Shock trial, we have spent over 2 decades adopting standardized protocols focused on early recognition, rapid intervention, and the expansion of our mechanical circulatory support toolkit. Despite these efforts, the survival curve for cardiogenic shock (CS) remains frustratingly flat.^1^ In a landscape long defined by neutral or inconclusive evidence, the DanGer Shock trial stands as a pivotal randomized controlled trial that demonstrated a clear mortality benefit with the use of a microaxial flow pump for acute myocardial infarction (AMI)-associated CS. Although these results are encouraging, they are also sobering: mortality in the Impella arm remained at an unacceptably high 45.8%, compared to 58.5% in the standard-care group.^2^ Although the use of Impella across the United States is expected to increase, several barriers to its widespread adoption remain. High acquisition costs, a relatively steep learning curve for operators, and the need for intensive postimplant management limit its availability. These challenges will restrict this life-saving technology to high-volume centers, creating a gap in access for patients at community hospitals.

Modi et al^3^ address this important issue in their paper, evaluating the outcome differences in patients who receive an Impella for CS at the index hospital vs those who require transfer to an Impella-capable hospital. The authors used the National Inpatient Sample database from 2016 to 2021 and identified 11,264 hospitalizations in which an Impella was used for CS to compare outcomes between these 2 groups. Only 16% of the patients in this cohort had AMI as the primary diagnosis. Minor differences in baseline characteristics were observed, with transferred patients being slightly younger (age 63.5 vs 64.6 years; *P* < 0.001). Approximately 28% of the cohort was transfers, most of whom were managed at large, urban teaching hospitals. This is expected, as these institutions function as “Shock Hubs” offering specialized shock teams with the capability to escalate and manage mechanical circulatory support devices. Transferred patients were significantly less likely to receive an Impella in the first 24 hours of admission and faced delays in device placement. We observed a similar pattern in another analysis of patients receiving an Impella or other temporary left ventricular assist devices for ST-segment elevation myocardial infarction–related CS.^4^ These delays likely reflect the additional time needed to reassess patients after transfer.

Although the study demonstrated no significant difference in mortality between the 2 groups, the potential for survival bias among transferred patients needs careful consideration, as acknowledged by the authors. Transferred patients had higher rates of complications, including acute kidney injury, a need for renal replacement therapy, limb ischemia, and blood transfusions. These findings reflect a more prolonged shock state before transfer. In addition, the nearly 2-fold higher use of extracorporeal membrane oxygenation within 24 hours of transfer suggests that many of these patients presented in more advanced stages of shock, where an Impella was potentially utilized as a left ventricular unloading strategy during extracorporeal membrane oxygenation support. These patterns emphasize the need to identify and triage high-risk patients earlier.

CS management requires a granular dissection of its phenotype and subsequent management; however, this level of detail is not possible with International Classification of Disease-coded administrative data. This remains the primary limitation of this study. “Door-to-support time” or “time to unloading” is crucial in the management of patients with CS and the inability to determine the timing of shock onset or the use of vasopressors makes interpretation challenging. As the authors acknowledge, these limitations should be considered when interpreting the study's findings.

## Future directions

### Systems-level approaches to CS

We have made significant progress in managing CS. Although this has not yet led to a drastic reduction in mortality, our understanding of the disease and our mechanical support tools have improved. The emergence of dedicated shock teams has enhanced the coordination and delivery of care, with several studies suggesting improved clinical outcomes.^5^^,^^6^ This team-based approach has also been shown to result in lower overall utilization of mechanical circulatory support while promoting more appropriate device selection.^7^ Given the complexity of this condition, it is difficult for every hospital to have a dedicated shock team.^8^ Therefore, the “hub-and-spoke” model—focused on early recognition and rapid transfer to specialized centers—will likely be the primary strategy moving forward.^9^

### Insights from established care models

The success of “door-to-balloon” targets for ST-elevation myocardial infarction provides a clear roadmap for CS care. By using standardized protocols, rapid diagnosis, and predefined transfer pathways, hospitals have significantly improved care for patient with acute coronary syndromes.^10^ A similar regional framework could work for shock, including early shock team activation and clear criteria for when to start mechanical support or transfer a patient. For patients who cannot be transferred, remote consultations and expert guidance from larger centers may help support management at the presenting institution.^9^ Finally, emerging data suggest that Black patients receiving mechanical circulatory support for AMI-related shock are transferred less often than their White counterparts, highlighting persistent disparities in access to advanced therapies and the need for more equitable regional shock systems.^11^

## Funding support and author disclosures

The authors have reported that they have no relationships relevant to the contents of this paper to disclose.
